# Supplementary material for: High-Density Lipoprotein Biomimetic Inorganic–Organic Composite Nanosystem for Atherosclerosis Therapy
Source: Polymers (Basel). 2025 Feb 26;17(5):625. doi: 10.3390/polym17050625 (PMC11902788; doi:10.3390/polym17050625)
Supplement: Supplementary file 1 [file polymers-17-00625-s001.zip › polymers-3469677-supplementary.pdf]

Supporting Information

High-density lipoprotein biomimetic inorganic organic composite nanosystem for atherosclerosis therapy

Yunpeng Zhang[a,b,c,†], Danni Liu[a,b,c,†],Yaoqi Wang[a,b,c], Qi Sun[a,b,c], Dong Mei[c,d], Xiaoling Wang[c,d], Yan Sun[c,e], Siyu Liu[a,b,c], Chunying Cui[a,b,c,*], Shuang Zhang[a,b,c,*]

[a]*School of Pharmaceutical Sciences, Capital Medical University, Beijing 100069, China*

[b]*Engineering Research Center of Endogenous Prophylactic of Ministry of Education of China, Beijing Area Major Laboratory of Peptide and Small Molecular Drugs, Beijing Laboratory of Biomedical Materials, Beijing 100069, China*

[c]*Laboratory for Clinical Medicine, Capital Medical University, Beijing 100069, China*

[d]*Department of Pharmacy, Beijing Children's Hospital, Capital Medical University, National Center for Children's Health, Beijing 100045, China*

[e]*Medical Oncology Department, Pediatric Oncology Center, Beijing Children's Hospital, Capital Medical University, National Center for Children's Health, Beijing 100045, China*

[†]Yunpeng Zhang and Danni Liu contributed equally to this work and shared the first authorship

[*]Corresponding authors: Tel./fax: +86 10 8391 1673 (Shuang Zhang), +86 10 8391 1668 (Chunying Cui);

E-mail addresses: zshuang@ccmu.edu.cn (Shuang Zhang), ccy@ccmu.edu.cn (Chunying Cui);

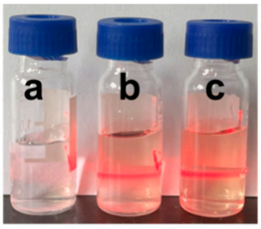

a. PBS
b. PEG-Ce/Mn
c. Apo-Ce/Mn

Figure S1. The Tyndall effect of PEG-Ce/Mn and Apo-Ce/Mn.

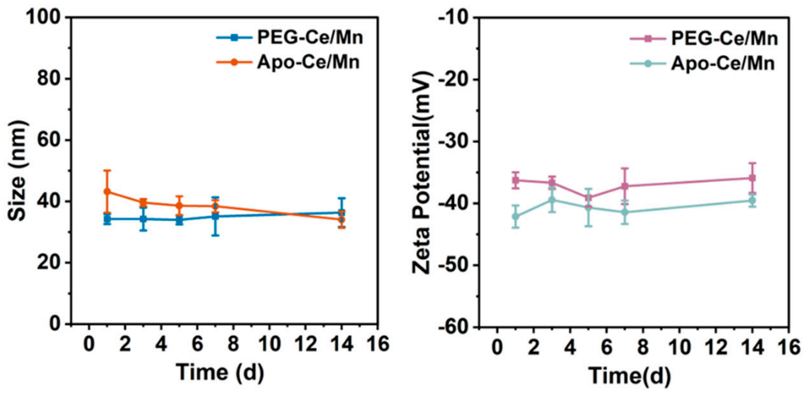

Figure S2. Long-term particle size and potential. (A)Average particle size of PEG-Ce/Mn and Apo-Ce/Mn at 1, 3, 5, 7, and 14 d. (B) Average zeta potential of PEG-Ce/Mn and Apo-Ce/Mn at 1, 3, 5, 7, and 14 d. n=3.

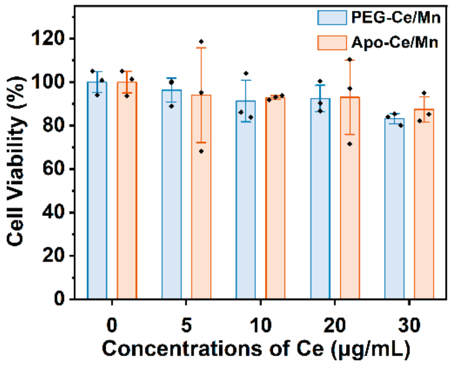

Figure S3. *In vitro* cell viability of RAW264.7 cells after 48 h of exposure to PEG-Ce/Mn and Apo-Ce/Mn at different concentrations. n=3.

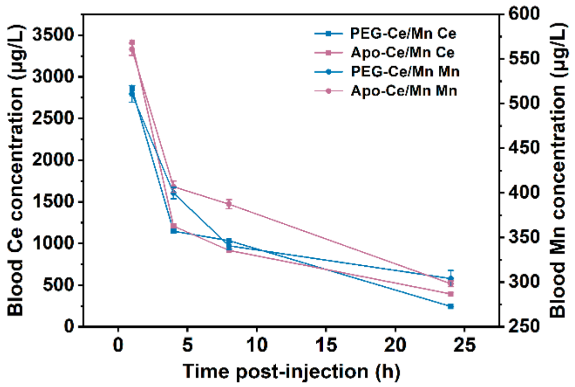

Figure S4. Concentrations of Ce and Mn in the blood of ApoE-/- mice at different time points after intravenous injection of PEG-Ce/Mn and Apo-Ce/Mn. n=3.

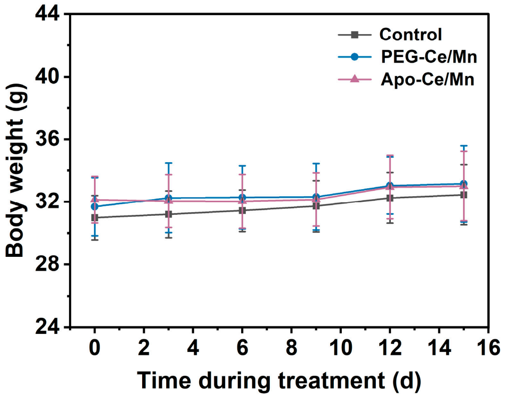

Figure S5. Body weight of ApoE-/- mice during treatment. n=6.

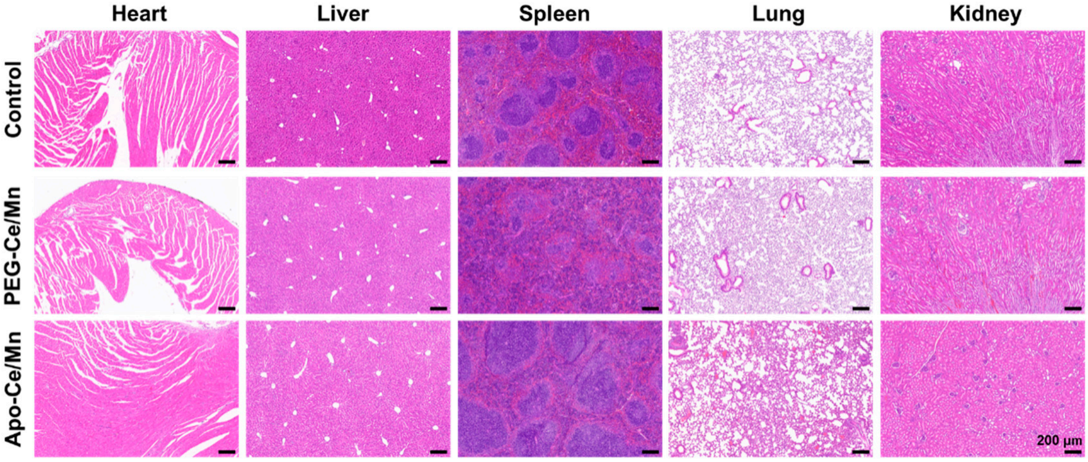

Figure S6. HE staining of major organs.
